# Bupi Yishen formula attenuates kidney injury in 5/6 nephrectomized rats via the tryptophan-kynurenic acid-aryl hydrocarbon receptor pathway

**DOI:** 10.1186/s12906-021-03376-1

**Published:** 2021-08-10

**Authors:** Yenan Mo, Xina Jie, Lixin Wang, Chunlan Ji, Yueyu Gu, Zhaoyu Lu, Xusheng Liu

**Affiliations:** 1 The Second Clinical Medical College, Guangzhou University of Chinese Medicine, Guangzhou, 520120 China; 2 Nephrology Department, the Second Affiliated Hospital of Guangzhou University of Chinese Medicine, Guangdong Provincial Hospital of Chinese Medicine, Guangzhou, 520120 China

**Keywords:** Chronic kidney disease, Bupi Yishen formula, Metabolomics, Kynurenic acid, Aryl hydrocarbon receptor pathway

## Abstract

**Background:**

Bupi Yishen Formula (BYF), a patent traditional Chinese medicine (TCM) formulation, has been used in the clinical treatment of chronic kidney disease (CKD). However, the mechanism of action of BYF has not been fully elucidated.

**Method:**

To investigate the variation in the metabolic profile in response to BYF treatment in a rat model of 5/6 nephrectomy (Nx), rats in the treatment groups received low- or high-dose BYF. At the end of the study, serum and kidney samples were collected for biochemical, pathological, and western blotting analysis. Metabolic changes in serum were analyzed by liquid chromatography-tandem mass spectrometry.

**Results:**

The results showed that BYF treatment could reduce kidney injury, inhibit inflammation and improve renal function in a dose-dependent manner. In total, 405 and 195 metabolites were identified in negative and positive ion modes, respectively. Metabolic pathway enrichment analysis of differential metabolites based on the Kyoto Encyclopedia of Genes and Genomes database identified 35 metabolic pathways, 3 of which were related to tryptophan metabolism. High-dose BYF reduced the level of kynurenic acid (KA) by more than 50%, while increasing melatonin 25-fold and indole-3-acetic acid twofold. Expression levels of aryl hydrocarbon receptor (AhR), Cyp1A1, and CyP1B1 were significantly reduced in the kidney tissue of rats with high-dose BYF, compared to 5/6 Nx rats.

**Conclusion:**

BYF has a reno-protective effect against 5/6 Nx-induced CKD, which may be mediated via inhibition of the tryptophan-KA-AhR pathway.

**Supplementary Information:**

The online version contains supplementary material available at 10.1186/s12906-021-03376-1.

## Background

Chronic kidney disease (CKD) is an emerging epidemic. The overall prevalence of which increased by 29.3% worldwide between 1990 and 2017. In total, it affected 697.5 million people in 2017, for a global prevalence of 9.1%, and is associated with high morbidity and mortality [1]. CKD results in changes in kidney structure and function, eventually leading to end-stage renal disease that requires dialysis or kidney transplantation. Thus, there is an urgent need for effective methods to prevent CKD progression.

In recent years, there has been increasing evidence regarding the beneficial effect of traditional Chinese medicine (TCM) on CKD in humans [2–4] and animals [5–7]. Bupi Yishen Formula (BYF) is a patent TCM formulation modified from the historical TCM prescription, Si-jun-zi Decoction. It was developed based on the results of text mining of medical records from Guangdong Provincial Hospital of Chinese Medicine, a tertiary hospital in southern China. BYF contains nine herbal medicines, including *Astragalus mongholicus* (Huangqi), *Codonopsis pilosula* (Dangshen), *Atractylodes macrocephala* (Baizhu), *Poria cocos* (Fuling), *Dioscorea opposita* (Shanyao), *Coici semen* (Yiyiren), *Polygonum multiflorum* (Heshouwu), *Cuscuta Chinensis* (Tusizi), and *Salvia miltiorrhiza* (Danshen). *Astragalus mongholicus* (Huangqi) and *Salvia miltiorrhiza* (Danshen), a commonly used drug pair for clinical treatment of CKD in traditional Chinese medicine with good efficacy, markedly reduced serum creatinine and urea nitrogen, and ameliorated tubular atrophy and interstitial fibrosis in CKD rats [7]. Our previous study observed that the major compound of *Astragalus membranaceus* (Huangqi), Astragaloside IV, prevented indoxyl sulfate-induced tubulointerstitial injury in mice via attenuation of oxidative stress [8]. *Codonopsis pilosula* (Dangshen) showed its renoprotective effect against renal ischemia/reperfusion in rats by inhibiting the proinflammatory cytokine TNF-α release [9]. *Atractylodes macrocephala* (Baizhu) could increase the level of superoxide dismutase (SOD), decrease the productions of IL-6 and TNF-α, and improve the renal tissue injury on nephrotic syndrome in rats [10]. *Poria cocos* (Fuling) ameliorated cisplatin-induced kidney tubular epithelial cells injury by inhibiting JNK, ERK, p38, and caspase-3 [11]. *Dioscoreaop posita* (Shanyao) could attenuate oxidative stress and fibrosis, regulate lipid metabolism, and inhibit inflammation against renal damage [12, 13]. The natural compound of *Polygonum multiflorum* (Heshouwu) played a protective role in ameliorating the progression of focal segmental glomerulosclerosis in a mouse model via activation of the Nrf2-Keap1 antioxidant pathway [14]. In our previous work, chemical constituents of BYF were systematically investigated by ultra-high-performance liquid chromatography (UHPLC) with linear ion trap-orbitrap mass spectrometry (MS) and UHPLC with triple-quadrupole tandem MS methods, which provided comprehensive qualitative and quantitative information for analysis of the main components of BYF. Eighty-six compounds, including flavones, phenolic acids, saponins, and other compounds, were identified [15]. Though we have not yet tested the effect of BYF in animals or cells, the major components of BYF have been shown reno-protective effect. Moreover, we have performed a multi-center, double-blind, randomized controlled trial to assess the efficacy and safety of BYF for delaying progression in patients with non-diabetes stage 4 CKD **(HERBAAL trial)** [16]. The result demonstrated that the BYF group experienced slower renal function decline compared to the losartan group over 48 weeks, without significant group differences in the incidence rates of adverse events [17]. However, the underlying mechanism of the effect of BYF on CKD is unclear.

Metabolomics is an evolving research area, with numerous successes in terms of characterizing biochemical metabolites related to disease progression, as well as responses to therapeutic interventions [18] . Therefore, metabolomics has been widely used to evaluate the efficacy and potential mechanisms of action of TCM prescriptions and herbs [7, 19, 20], thus facilitating the modernization of TCM. 5/6 nephrectomy (Nx) is the most classic CKD model as the glomerulosclerosis and tubulointerstitial fibrosis that develop after the 5/6 nephrectomy have been generally considered to represent the adverse consequences of a severe reduction in the number of nephrons [21].

In this study, we aimed to explore the beneficial effects of BYF on CKD, as well as its potential mechanisms of action, in 5/6 nephrectomized (Nx) rats. The characteristics of CKD and effects of BYF treatment were evaluated according to blood biochemical indexes and renal pathological changes. An untargeted metabolomics method, liquid chromatography-mass spectrometry/mass spectrometry (LC-MS/MS), was used for metabolic profiling, to investigate the response to BYF treatment of 5/6Nx CKD rats. Signaling pathways were examined by western blotting. Our findings validate the efficacy and mechanism of BYF, which may offer a promising approach for treatment of CKD in clinical practice.

## Methods

### Extract preparation and chemical analysis of BYF extract

The medicinal materials used to produce BYF were purchased from Kangmei Pharmaceutical Co., Ltd. (Guangdong, China). Information regarding components of BYF (e.g., Chinese, Latin and English names, medical parts, and places of origin) is shown in Suppl. Table 1. The nine herbs were mixed at the prescribed ratios, extracted three times (60 min) with boiling water (1:8), and filtered with gauze. The filtered BYF extract was obtained by solvent evaporation in a vacuum at 56 °C.

High-performance liquid chromatography (HPLC) was performed to verify the similarity of the major compounds in BYF prepared in this study and our previous study, in which the chemical constituents of BYF were systematically investigated by HPLC. The HPLC method is described in Suppl. Item 1.

### Experimental animals and medicinal intervention

Male Sprague–Dawley rats (specific pathogen-free grade; weight, 180–220 g) were purchased from the Laboratory Animal Center of Southern Medical University (Guangzhou, China). The rats were housed in a specific pathogen-free animal breeding room in Guangdong Provincial Hospital and given free access to water. The rats were fed according to a 12-h light/dark cycle. All experiments were evaluated and approved by the Ethics Committee of Animal Experiments, Guangdong Provincial Hospital of Chinese Medicine (approval no. 2019026).

**Fig. 1:**
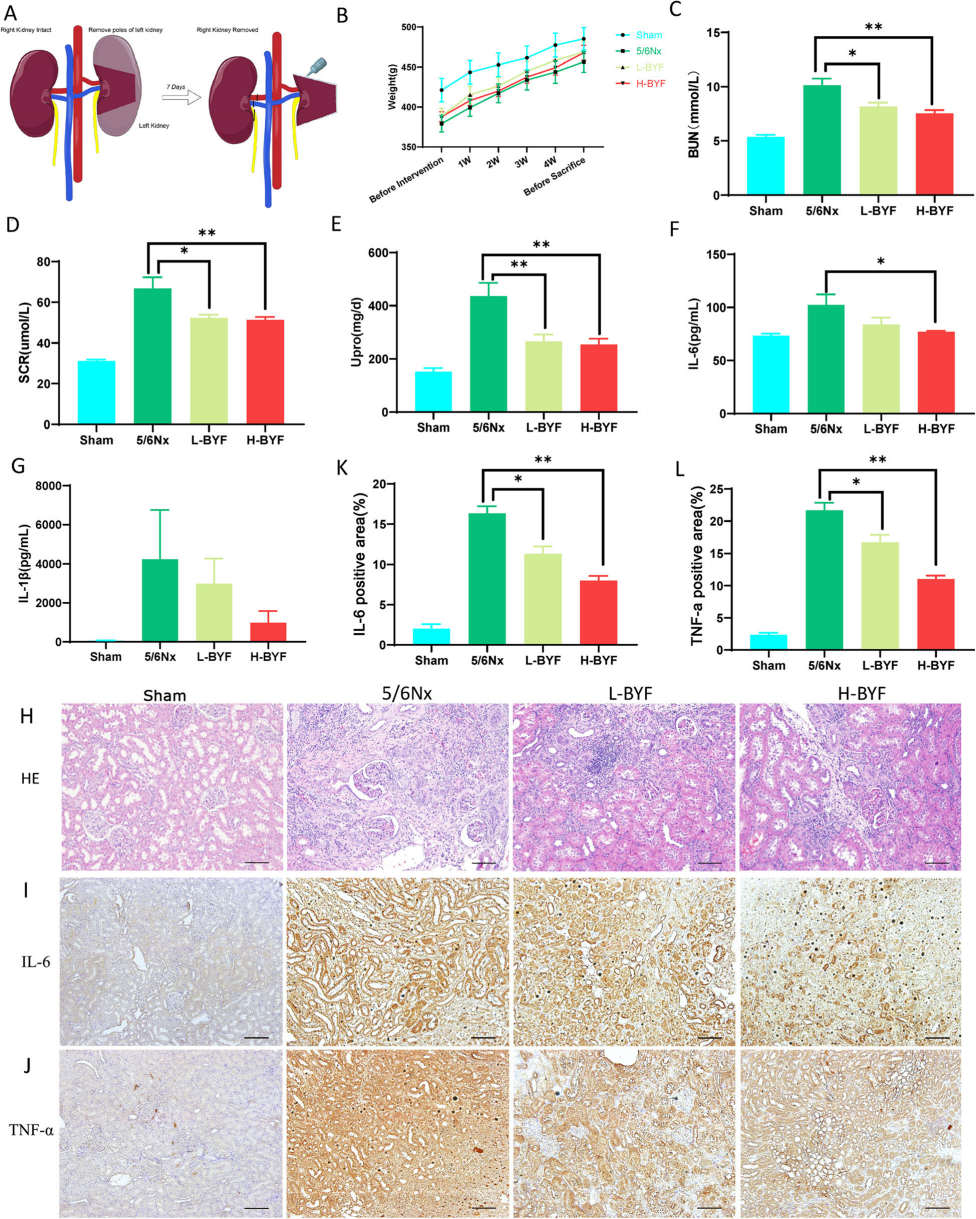
Effects of BYF on 5/6Nx CKD rats. **(A)** 5/6Nx method. **(B)** Body weight. **(C)** Blood urea nitrogen. **(D)** Serum creatinine. **(E)** Twenty-four-hour urinary protein quantitation. **(F)** Serum IL-6. **(G)** Serum IL-1β. **(H)** Hematoxylin and eosin staining of kidney tissues. **(I-K)** Immunohistochemical staining of TNF-α and IL-6 expression in kidney. Data are presented as the means ± standard error of the mean. *n* = 12 rats per group in the 5/6Nx, L-BYF, and H-BYF groups; *n* = 10 in the sham group (*P < 0.05, **P < 0.001)

Forty-six rats were randomly divided into the sham group (*n* = 10) and the CKD group (*n* = 36). CKD was induced by 5/6Nx in rats as follows. All rats were anesthetized with an intraperitoneal injection of 2.0% pentobarbital sodium (30 mg/kg body weight). Two-thirds of the left kidney was initially removed. Seven days later, total right Nx was performed. Rats in the sham group only underwent removal of the fat sac, without Nx **(**Fig. 1A**)**.

Four weeks after surgery, the CKD group was randomly divided into the 5/6Nx, L-BYF, and H-BYF groups, according to the random number table method. BYF-treated rats received low-dose BYF (3.2 g/kg.w) or high-dose BYF (6.4 g/kg.w) by intragastric administration, once daily. Based on the clinical usage [17] and the Meeh-Rubner equation [22] of dose conversion, 3.2 g/kg and 6.4 g/kg dosage was chosen for low-dose BYF and high-dose BYF, respectively.

Rats in the sham and 5/6Nx groups received an identical volume of normal saline. Body weight was recorded once weekly throughout the experiment. BYF intervention lasted for four weeks. At the end point of the experiment, rats were anesthetized with an intraperitoneal injection of 2.0% pentobarbital sodium (30 mg/kg body weight). When rats became unconscious, they were euthanized using cervical dislocation. Blood and kidney tissues were collected and processed for untargeted metabolomic, histological, and western blotting analyses.

### Biochemical analysis and enzyme-linked immunosorbent assay

Serum creatinine and blood urea nitrogen were measured by Cobas C702 automatic analyzers (Roche, Basel, Switzerland). Proteinuria was measured using a bicinchoninic acid protein detection kit (Thermo Fisher Scientific, Waltham, MA, USA). Serum levels of interleukin (IL)-6 and IL-1β were measured in accordance with the enzyme-linked immunosorbent assay kit instructions (R&D Systems, Minneapolis, MN, USA).

### Histological analysis

The kidney tissues of rats were fixed with paraformaldehyde, and then dehydrated and embedded in paraffin. Paraffin-embedded tissues were cut into 3-μm sections and stained with hematoxylin and eosin (Boster Bio, Wuhan, China).

### Immunohistochemistry

Paraffin-embedded rat kidney slides were deparaffined, rehydrated, and immersed in 3% hydrogen peroxide for 10 min at room temperature to block endogenous peroxidase activity. All sections were heated in Tris-EDTA buffer (pH 9.0, Boster, Wuhan, China), blocked with 5% blocking buffer for 30 min at 37 °C, incubated with primary antibodies against Tumor necrosis factor (TNF-α) (1:100, Abcam Cat# ab6671, Cambridge, England), IL-6 (1:100, Abcam Cat# ab9324, Cambridge, England) at 4 °C overnight, incubated with species-specific secondary antibody (SV0004, Boster, Wuhan, China), developed with 3,3′-diaminobenzine (DAB, Invitrogen, California, USA) and counterstained with hematoxylin. The integrated optical density (IOD) values of the positive staining areas were measured by ImagePro Plus 6.0 software (Media Cybernetics, CA, USA).

### Sample preparation and LC-MS/MS analysis for metabolomics

Sample preparation: 100-μL samples were extracted by direct addition of 300 μL of precooled methanol and acetonitrile (2:1, v/v). Internal standard mixes 1 and 2 were added for sample preparation quality control (QC). After samples had been vortexed for 1 min and incubated at − 20 °C for 2 h, they were centrifuged at 4000 rpm for 20 min. The supernatants were subjected to vacuum freeze-drying. The dried metabolites were resuspended in 150 μL of 50% methanol for 30 min and centrifuged at 4000 rpm. The supernatants were then transferred to sample vials for LC-MS/MS analysis. A QC sample was prepared by blending the same volume of each sample to evaluate the reproducibility of the overall LC-MS/MS analysis, as described in Suppl. Item 2.

Global metabolomics analysis by LC-MS/MS: Samples were analyzed on a Waters 2D UHPLC column (Waters, Milford, MA, USA), coupled to a Q-Exactive mass spectrometer (Thermo Fisher Scientific) using a heated electrospray ionization source, controlled by Xcalibur 2.3 software (Thermo Fisher Scientific). Chromatographic separation was carried out on a Waters ACQUITY UHPLC BEH C18 column (1.7 μm, 2.1 mm × 100 mm; Waters), with the column temperature maintained at 45 °C. The normal mobile phase was 0.1% formic acid (A) and acetonitrile (B) in positive ion mode. In negative ion mode, the mobile phase was 10 mM ammonium formate (A) and acetonitrile (B). The gradient conditions were as follows: 0–1 min, 2% B; 1–9 min, 2% B to 98% B; 9–12 min, 98% B; 12–12.1 min, 98% B to 2% B; and 12.1–15 min, 2% B. The flow rate was 0.35 mL/min, and the injection volume was 5 μL.

The mass spectra of the positive/negative ion modes were captured using the following settings: spray voltage, 3.8/− 3.2 kV; gas flow rate in sheath, 40 arbitrary units; aux gas flow rate, 10 arbitrary units; aux gas heater temperature, 350 °C; and capillary temperature, 320 °C. The full scanning range was 70–1050 m/z with a resolution of 70,000, and the automatic gain control target for MS acquisitions was set to 3e6 with a maximum ion injection time of 100 ms. The top three precursors were selected for subsequent MS/MS fragmentation with a maximum ion injection time of 50 ms and resolution of 17,500, using an automatic gain control of 1e5. The stepped normalized collision energies were set to 20, 40, and 60 eV. Nitrogen was used as atomizer and auxiliary gas. The serum samples were analyzed in positive and negative ion modes, and the scanning mass-to-charge (m/z) range was 50 to 1500 Da.

To analyze metabolomic data, the mass spectrum data were processed for noise reduction, peak alignment, and peak identification. Peak intensities were normalized using internal standards. Differential metabolites were analyzed using partial least square-discriminant analysis. Differential metabolites among groups were characterized by a variable importance value > 1, fold-change ≥1.2 or ≤ 0.83, and q-value < 0.05. By comparison with databases including ChemSpider (www.chemspider.com) and HMDB (www.hmdb.ca), differential metabolites were preliminarily identified based on the mass fragmentation patterns. Pathway analysis was performed using the Kyoto Encyclopedia of Genes and Genomes (KEGG) database.

### Western blotting analysis

Kidney tissues were lysed in 1 mL radioimmunoprecipitation assay lysis buffer containing 1 mM phenylmethyl sulfonyl fluoride and 1% phosphatase inhibitor cocktail (Thermo Fisher Scientific). A bicinchoninic acid protein detection kit was used to detect protein concentrations. Protein samples (50 μg) were boiled with sodium dodecyl sulfate polyacrylamide gel electrophoresis loading buffer, then electrophoresed on a 10% polyacrylamide gel under denaturing conditions and wet-transferred to polyvinylidene difluoride membranes (Millipore, Burlington, MA, USA). Membranes were exposed to blocking buffer for 2 h and hybridized with primary antibody against aryl hydrocarbon receptor (AhR) (1:500; Abcam, Cat# ab84833 Cambridge, UK), CYP1A1 (1:400; Santa Cruz Biotechnology, Cat# sc-25304, CA, USA), CyP1B1 (1:6000; Abcam, Cat# ab185954, Cambridge, UK), or β-actin (1:2000; Cell Signaling Technologies, Cat# 4970S, Boston, USA) overnight at 4 °C, followed by horseradish peroxidase-labeled anti-rabbit IgG (1:3000, Cell Signaling Technologies, Cat# 7074S, Boston, USA) or anti-mouse IgG (1:3000, Cell Signaling Technologies, Cat# 7076S, Boston, USA) at room temperature. Membranes were washed and then visualized using an enhanced chemiluminescence detection system (Bio-Rad, Hercules, CA, USA). The signals were captured and analyzed using Image Lab System (Bio-Rad).

### Statistical analysis

SPSS software (version 18.0, SPSS, Inc., Chicago, IL, USA) was used for statistical analysis. All data with normal distributions are presented as the mean ± standard error of the mean. Differences between groups were determined by one-way analysis of variance, followed by the Tukey test. Differences were considered statistically significant at *p* < 0.05.

## Results

### QC of BYF

HPLC indicated that the major components in BYF were similar between this study and the previous study, as shown in Suppl. Fig. 1.

### BYF alleviated renal failure phenotypes in CKD rats

We first evaluated the protective effect of BYF on CKD. It is common for patients with CKD to experience weight loss. Notably, L-BYF and H-BYF appeared to promote weight gain in CKD rats compared to 5/6Nx rats, although weight did not significantly differ among groups **(**Fig. 1B**)**. Blood urea nitrogen, serum creatinine, and 24-h proteinuria levels were significantly higher in the 5/6Nx group than in the sham group. These levels decreased in the BYF-treated groups in a dose-dependent manner **(**Fig. 1C-E**)**. With regard to the systematic inflammatory response, serum IL-6 and serum IL-1β were elevated in 5/6Nx rats compared to sham rats, and BYF treatment significantly decreased the serum IL-6 and tended to reduce the serum IL-1β **(**Fig. 1F, G**)**.

Consistent with the improved renal function and inhibited inflammation, histopathological changes were improved by low- and high-dose BYF treatments. Hematoxylin and eosin staining of renal tissues showed obvious mononuclear lymphocyte infiltration, enlargement of the renal tubular lumen, interstitial fibrosis, and renal tubular atrophy in the 5/6Nx group **(**Fig. 1H**)**. Compared to sham rats, the expression of IL-6 and TNF-α in kidneys were elevated in 5/6Nx rats and BYF significantly reduced kidney IL-6 and TNF-α **(**Fig. 1I-L**)**.

### Global metabolic profiling of serum metabolites in CKD rats

#### Assessment of data quality using LC-MS/MS

**Fig. 2:**
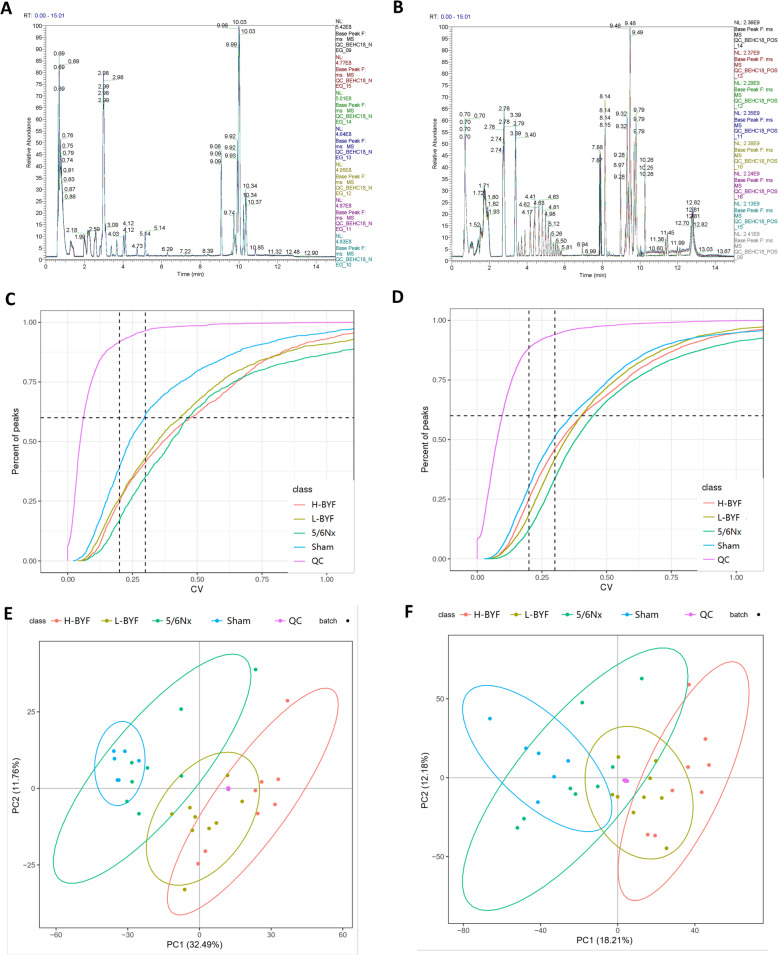
Assessment of data quality of the LC-MS/MS method. Overlapping total ion current chromatograms of QC samples indicated that the variations occurring during large-scale sample analysis were acceptable in negative ion **(A)** and positive ion **(B)** modes. Principle component analysis showed that all QC samples were clustered closely in negative ion **(C)** and positive ion **(D)** modes. The ratio between the number of compounds with < 3 0% coefficient of variation of the relative peak area in QC samples and all compounds detected was greater than 60% in both negative ion **(E)** and positive ion **(F)** modes

Metabolomics assessment of serum samples from each group was performed with LC-MS/MS, in both positive and negative ion modes. The overlapping total ion current chromatograms of QC samples indicated that the variations during large-scale sample analysis were acceptable **(**Fig. 2A, B**)**. To ensure that the LC-MS system was stable, principle component analysis was performed, which revealed that all QC samples were consistently clustered together **(**Fig. 2C, D**)**. The ratio between the number of compounds with < 30% coefficient of variation of the relative peak area in QC samples and all compounds detected was greater than 60% **(**Fig. 2E, F**)**. These results indicated that the LC-MS method was repeatable and stable, and thus suitable for further analysis.

#### Metabolic profiles of 5/6Nx and H-BYF rats

**Fig. 3:**
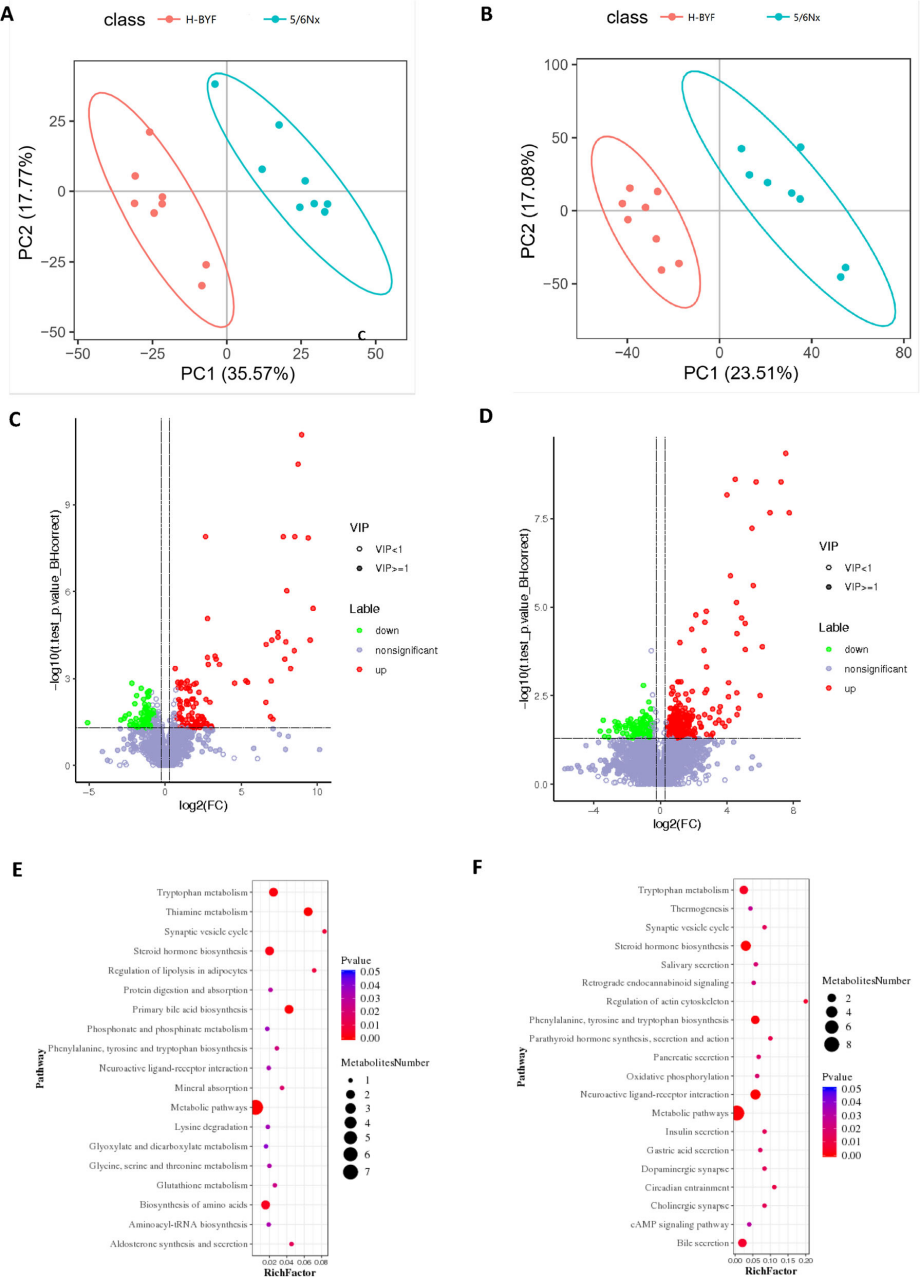
Metabolic profile differences between the 5/6Nx and H-BYF groups. Principle component analysis score plots of serum metabolites: comparison between the 5/6Nx and H-BYF groups in negative ion **(A)** and positive ion **(B)** modes. Volcano plot for differential metabolites between the 5/6Nx and H-BYF groups in negative ion **(C)** and positive ion **(D)** modes. Bubble plots of KEGG pathway enrichment analysis between 5/6Nx and H-BYF groups in negative ion **(E)** and positive ion **(F)** modes

To evaluate the metabolic changes in CKD rats, principle component analysis was used. The initial metabolic state of the 5/6Nx group was markedly different from that of the H-BYF group (Fig. 3A, B), which demonstrated that BYF treatment significantly altered the serum metabolic profile in CKD rats.

To further compare the metabolic changes between the 5/6Nx and H-BYF groups, partial least square-discriminant analysis was performed. Metabolites with a variable importance value > 1, fold-change ≥1.2 or ≤ 0.83, and q-value < 0.05 were defined as differential metabolites among the groups. In total, 405 and 195 metabolites with significant changes in peak intensity were detected in negative ion mode (123 upregulated and 72 downregulated metabolites, respectively) and positive ion mode (293 upregulated and 112 downregulated metabolites, respectively) **(**Fig. 3C, D**)**.

#### Metabolic pathway analysis of BYF effects on CKD rats

To explore the functional significance of the serum metabolic changes in the BYF group, metabolic pathway enrichment analysis of differential metabolites was carried out using the KEGG database. There were 35 metabolic pathways (20 and 23 metabolic pathways in negative and positive ion modes, respectively), including neuroactive ligand-receptor interaction, thiamine metabolism, steroid hormone biosynthesis, metabolic pathways, primary bile acid biosynthesis, tyrosine and tryptophan biosynthesis, tryptophan metabolism, phenylalanine-bile secretion, bile secretion, regulation of actin cytoskeleton, circadian entrainment, parathyroid hormone synthesis, secretion, and action, biosynthesis of amino acids, aldosterone synthesis and secretion, cholinergic synapse, dopaminergic synapse, insulin secretion, synaptic vesicle cycle, gastric acid secretion, pancreatic secretion, oxidative phosphorylation, salivary secretion, retrograde endocannabinoid signaling, glutathione metabolism, thermogenesis, cAMP signaling pathway, protein digestion and absorption, glycine, serine and threonine metabolism, aminoacyl-tRNA biosynthesis, mineral absorption, lysine degradation, phosphonate and phosphinate metabolism, taste transduction, glyoxylate and dicarboxylate metabolism, and inflammatory mediator regulation of tryptophan channels **(**Fig. 3E, F**)**.

#### BYF significantly inhibited tryptophan-KA-AhR pathway

**Fig. 4:**
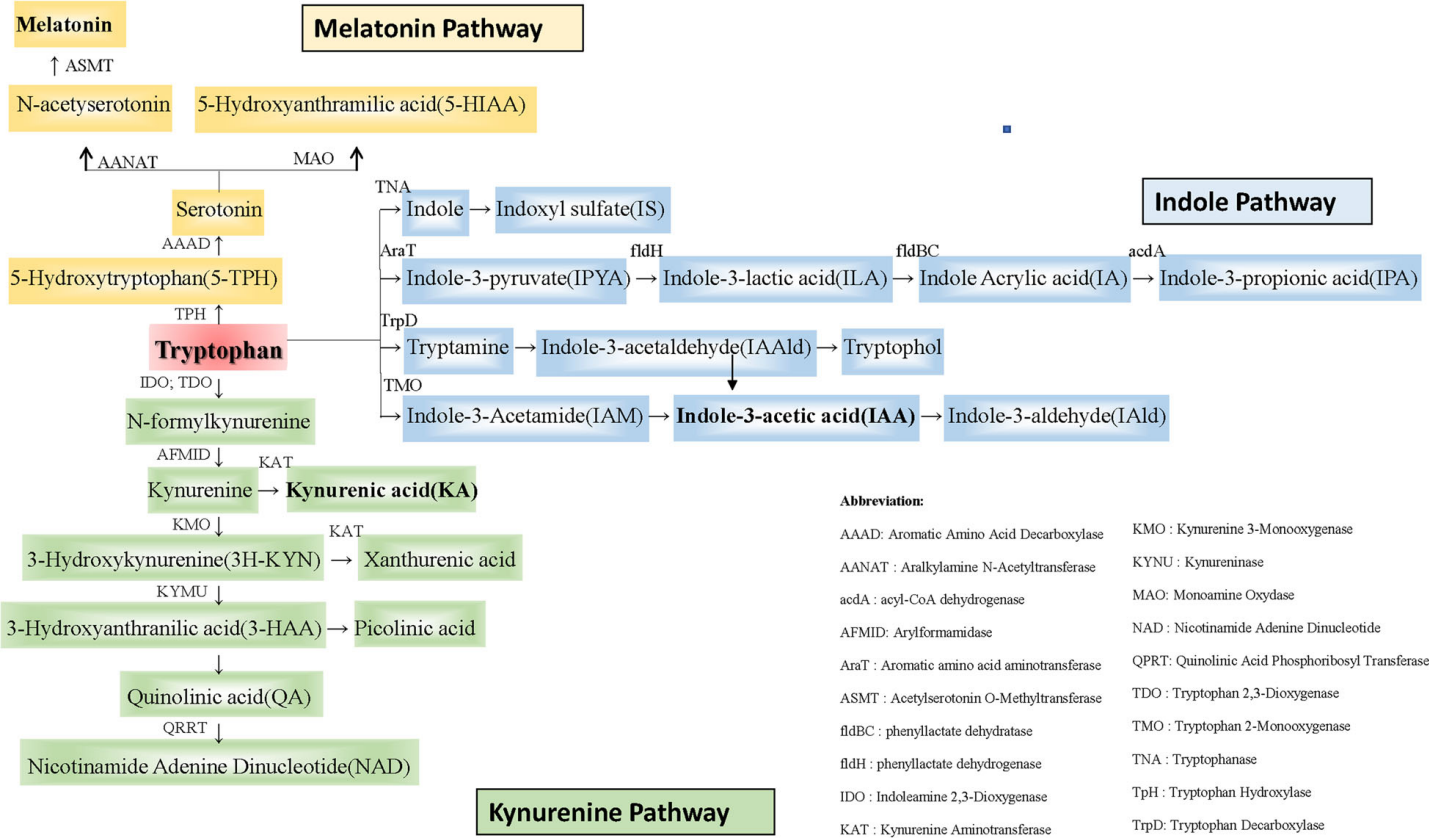
Tryptophan-KA-AhR pathway. Pathways of tryptophan metabolism through the indole, melatonin, and KA pathways

**Fig. 5:**
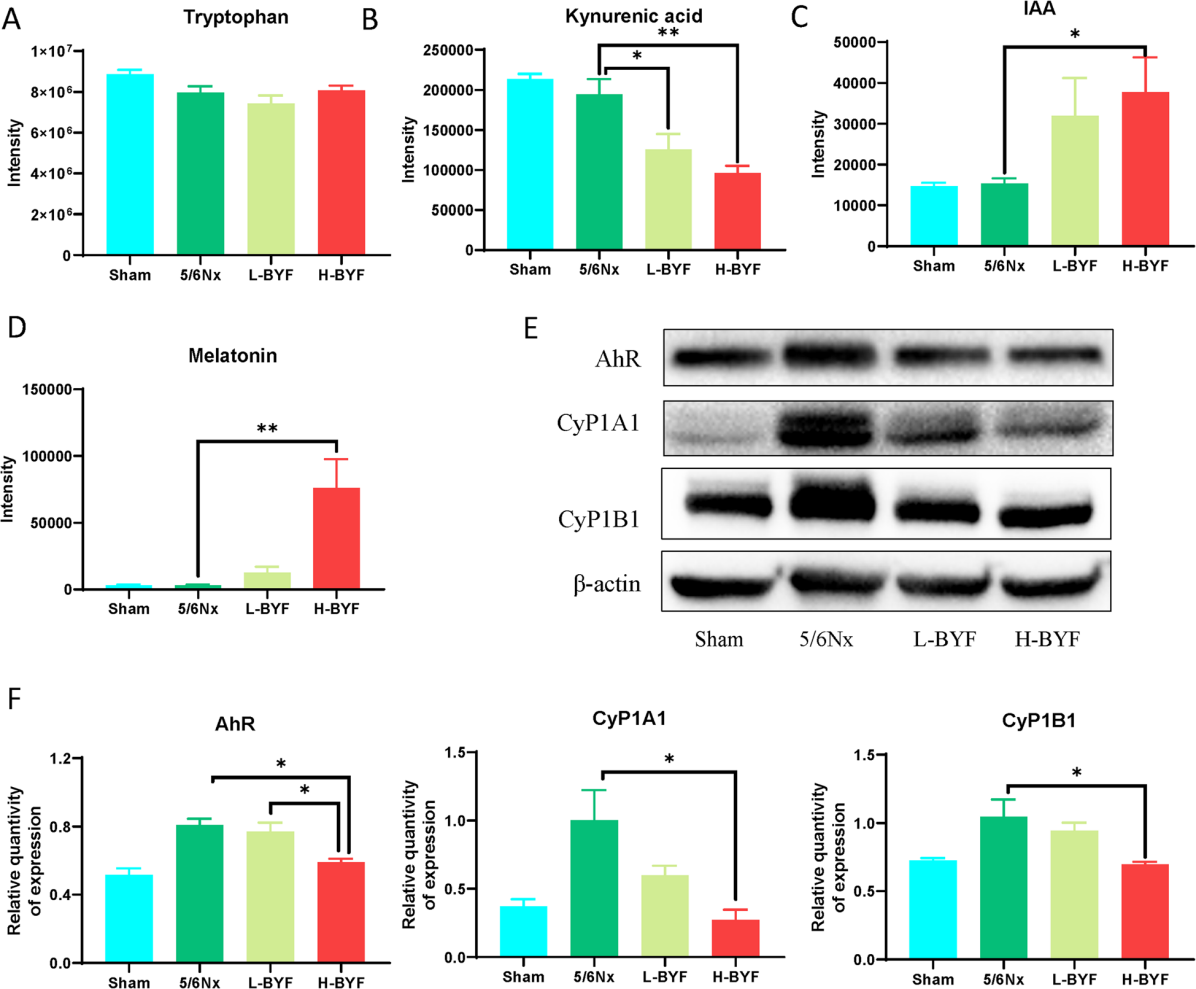
Tryptophan-KA-AhR pathway. BYF increased the production of kynurenic acid and melatonin, whereas it decreased the production of indole-3-acetic acid. The intensities of tryptophan **(A)**, kynurenic acid **(B)**, indole-3-acetic acid **(C)**, and melatonin **(D)** were measured by LC-MS/MS. **(E-F)** BYF inhibited the expression of the AhR signaling pathway. *n* = 3 per group (*P < 0.05, **P < 0.001)

Three of the thirty-five metabolic enrichment pathways were related to tryptophan metabolism. Tryptophan metabolism involves three pathways: indole, kynurenine, and serotonin **(**Fig. 4**)**. The level of tryptophan did not differ among groups. High-dose BYF treatment reduced the level of kynurenic acid (KA) by more than 50%, while increasing the level of melatonin 25-fold and the level of indole-3-acetic acid twofold **(**Fig. 5A-D**)**. KA is a type of uremic toxin produced from tryptophan and an endogenous ligand of the AhR. To determine whether BYF treatment influenced the AhR pathway in kidney tissues, the expression levels of AhR, CyP1A1, and CyP1B1 were measured by western blotting. The results showed that BYF treatment significantly inhibited AhR signaling in a dose-dependent manner **(**Fig. 5E**)**.

## Discussion

BYF treatment markedly improved kidney function, reduced proteinuria, inhibited the inflammatory response, and alleviated interstitial fibrosis and tubular atrophy in CKD rats in a dose-dependent manner. Metabolomics analysis indicated that BYF regulated tryptophan metabolism and reduced KA production. Expression levels of AhR, Cyp1A1, and CyP1B1 in kidney tissue were significantly reduced in rats that received BYF treatment compared to 5/6Nx rats. Therefore, BYF treatment may protect the kidney through inhibition of the tryptophan-KA-AhR pathway in CKD rats.

Because of the limited usefulness of angiotensin-converting enzyme inhibitors/angiotensin II receptor blockers in patients with advanced CKD, there is an urgent need for additional renal protective therapies. BYF was shown to slow renal function decline, compared to losartan, over 48 weeks in a multi-center, double-blind, randomized controlled trial [17]. In previous studies by our group, compounds contained in BYF were identified by UHPLC-MS. Specifically, 15 flavones, 10 saponins, 12 phenolic acids, and 49 other compounds were detected [15]. According to TCM theory, Qi deficiency and blood stasis (Qi-Xu-Xue-Yu) occur during the progression of CKD [23]. *Astragalus mongholicus* (Huangqi) are commonly combined to replenish Qi, while *Salvia miltiorrhiza* (Danshen) is often used to activate blood. The effects of Huangqi and/or Danshen on metabolic pathways in rat models of CKD and other diseases have been reported in previous studies [7, 24, 25]. Astragaloside IV is one of the main active ingredients of *Astragalus mongholicus* and is used as a quality control marker of *Astragalus mongholicus* (Huangqi) in the Chinese Pharmacopeia. Pharmacological effects of Astragaloside IV on renoprotection attributable to its anti-inflammatory, antioxidant, anti-apoptotic properties, and the roles in enhancement of immunity, are associated with multiple signaling pathways, including the AMPK signaling pathway, NF-κB signaling pathway, Nrf2 antioxidant signaling pathways and PKC-α-ERK1/2-NF-κB pathway [26]. On the other hand, salvianolic acid A in *Salvia miltiorrhiza* (Danshen) effectively protects the kidney against oxidative stress in 5/6Nx rats. One of the pivotal mechanisms for the protective effects of salvianolic acid A on kidney injury was mainly related with its antioxidative roles by activating the Akt/GSK-3β/Nrf2 signaling pathway and inhibiting the NF-κB signaling pathway [27]. Cardioprotective effect of rosmarinic acid in *Salvia miltiorrhiza* (Danshen) against myocardial ischaemia/reperfusion injury via suppression of the NF-κB inflammatory signalling pathway and ROS production in mice [28]. *Poria cocos* (Fuling) treatment inhibited the upregulation of IκB/NF-κB pathway and prevented the downregulation of cytoprotective Keap1/Nrf2 pathway [29]. Atractylenolide I in *Atractylodes macrocephala* (Baizhu) ameliorates sepsis syndrome by reduction of pro-inflammatory cytokines and LPS, and provides an improvement in liver and kidney functions [30]. Therefore, the chemical ingredients contained in the drug compound are complex, and its pharmacological effect is the comprehensive or integrated effect of the multiple active ingredients contained in the compound through multiple pathways, multiple links, and multiple targets, and the mechanism of action is complex.

In agreement with the holistic thinking of TCM, metabolomics has shown potential in bioactivity evaluation and action mechanism of TCM as well as pharmaceutical research and development. Compared with transcriptomics, proteomics, and microbiome, metabolomics has the following advantages: firstly, metabolomics amplifies small changes in gene and protein expression, making detection easier. Secondly, metabolomics researchers do not need to establish whole genome sequencing and a large number of expressed sequence tags databases, and the types of metabolites are much smaller than genes, proteins, and microorganisms in quantitative terms. Thirdly, the separation and culture of microorganisms is difficult to achieve the desired effect in microbiome, leading to difficulty in studying bacterial interactions in complex microbial communities, but the technology used in the metabolomics research is more versatile and easier to be accepted by researchers. Therefore, metabolomics has been applied to discover the mechanism of BYF protecting against CKD.

There was no statistically significant difference between the male and female CKD patients by gender subgroup analysis in our previous clinical RCT. Female rats have menstrual cycles and it is inconvenient to collect 24 h urine, so we chose male rats for this study. In our study, we identified more than 500 significantly different metabolites between H-BYF and 5/6Nx rats with LC-MS/MS-based nontargeted metabolomics. Metabolic pathway enrichment analysis of differential metabolites based on the KEGG database identified 35 metabolic pathways, 3 of which were related to tryptophan metabolism. As an essential aromatic amino acid, tryptophan is a biosynthetic precursor of a variety of host and microbial metabolites [31]. The metabolism of tryptophan has three major pathways in the gastrointestinal tract (Fig. 4A): 1) the intestinal microorganism-mediated indolic pathway, which transforms tryptophan into several molecules (e.g., indole-3-acetic acid, indole-3-aldehyde, indole-3-propionic acid, and indole-3-acetaldehyde) [32–34]; 2) the kynurenine pathway, which produces kynurenine and downstream products such as KA and quinolinic acid in both immune and epithelial cells via the rate-limiting enzyme, indoleamine 2,3-dioxygenase [35–37]; and 3) the serotonin pathway, which produces melatonin and serotonin in enterochromaffin cells via tryptophan hydroxylase-1 [38]. Indeed, tryptophan-derived uremic toxins, including KA and indole-3-acetic acid, are endogenous agonists of the AhR complex. Tryptophan-derived uremic toxins accumulate in patients with CKD and activate the AhR pathway [39], which results in pro-inflammatory, −oxidant, −apoptotic, and -coagulant effects [40].

Compared to our 5/6Nx rats, H-BYF rats produced more KA but less melatonin and indole-3-acetic acid. These findings indicated that BYF might inhibit the kynurenine pathway, while facilitating a shift toward the serotonin and indole pathways. KA is a stable end product of the kynurenine pathway, whereas kynurenine is not. Thus, KA is the most sensitive marker of kynurenine pathway activation, which is closely associated with the presence and/or development of kidney dysfunction/failure [41]. Lower kidney clearance of KA was associated with significantly greater risk of CKD progression compared to indoxyl sulfate [42]. Higher circulating concentrations of KA are associated with greater oxidative stress and incident myocardial infarction [43, 44]. A large proportion (95%) of tryptophan is metabolized through the kynurenine pathway, which generates considerably more metabolites than the other two pathways. Plasma concentrations of strongly protein-bound toxins are reportedly higher in patients with CKD than in healthy individuals (KA: 75-fold [45, 46], indoxyl sulfate: 72-fold [47, 48], and indole-3-acetic acid: 2.4-fold [48]). In addition, the 1-methyltryptophan led to an increase in the plasma levels of KA in pigs, of approximately 5 μM, which was sufficient to activate AhR because of the high affinity of KA to AhR, even at low micromolar concentrations [49]. Treatment of murine splenocytes with 5 μM KA exerted a slight proliferative effect, concurrent with increased secretion of IL-1β and IL-6. This finding suggested that KA exerts its biological effects via AhR [50]. We noted that IL-1β and IL-6 levels increased with BYF treatment, but not significantly (presumably due to the small sample size). Notably, H-BYF led to reduction of KA by half, while the level of indole-3-acetic acid increased twofold. This inhibited the AhR signaling pathway, reducing the expression levels of AhR, CyP1A1, and CyP1B1. These changes markedly reduced proteinuria and the inflammatory response, thus improving kidney function and histopathological changes in CKD rats.

Importantly, the production of melatonin was increased with BYF treatment. There is increasing evidence that altered circadian rhythms and serum melatonin levels are common in patients with CKD, and that the production of melatonin declines during the progression of CKD to end-stage renal disease [51]. Melatonin has shown a reno-protective effect in various renal injury animal models. For instance, melatonin suppressed the renin-angiotensin system in the kidney in 5/6Nx models [52], inhibited fibroblast-myofibroblast trans-differentiation during renal fibrosis in unilateral ureteral obstruction mice [53], and mitigated oxidative stress in diabetic nephropathy rats [54].

It has been reported that activated AhR aggravates renal damage and mediates CKD complications, including cardiovascular disease, anaemia, bone disorders, cognitive dysfunction and malnutrition, and that it influences drug metabolism in individuals with CKD [55]. There are two AhR signalling pathway. One is noncanonical AhR signalling, which controls AhR gene expression through non- xenobiotic-responsive element DNA-response elements. AhR signaling pathway also interacts with additional transcriptional factors such as STAT [56], Nrf2 [57], activator protein-1 [58], and NF-κB [59], by binding to them and modulating their target genes to enhance inflammatory response. In additionally, AhR in the cytoplasm can activate other cytoplasmic proteins, like Smads, β-catenin, MAPK family p38 [60], NADPH oxidase, and extracellular signal-regulated kinase. We have discussed the molecular mechanism of several compounds of BYF protecting against kidney injury, which are related to many signaling pathways including AMPK signaling pathway, NF-κB signaling pathway, Nrf2 antioxidant signaling pathways. However, how AhR interacts with or influences other transcriptional factors and cytoplasmic protein need further study. Integrating transcriptomics and metabolomics might be helpful to answer this question.

We are in an era where the need for novel approaches for delay CKD progression is on its all-time high. Chinese medicines provide a rich of resources for drug discovery and development. Despite the availability of our clinical RCT and present animal study of BYF treatment, there are still some inherent hurdles hinder their clinical translation, including that the oral bioavailability is weak [61] and the role of key components are difficult to determine. Microbial degradation in the gut is one of the most common and identified reasons for poor pharmacokinetic and oral bioavailability. The intestinal flora is involved in the metabolism of nutrients and food, and plays a core role in the conversion of original herbal medicine components into functional metabolites. Instead of screening functional ingredients directly from herbal extracts, to study the effect of Chinese medicine and gut microbiota using multi-omics approaches seems critical [62].

## Conclusion

In our study, an LC-MS/MS-based nontargeted metabolomics approach was used to investigate the reno-protective effects and mechanism of action of BYF in 5/6Nx CKD rats. Treatment with BYF alleviated kidney injury, improved renal function, and partially reversed metabolic abnormalities. Metabolomics analysis indicated that BYF regulated tryptophan metabolism and reduced KA production, while reducing the expression levels of AhR, CyP1A1, and CyP1B1 in kidney tissue. Therefore, we presume that BYF treatment protected the kidney through inhibition of the tryptophan-KA-AhR pathway in CKD rats.

## Supplementary Information

**Additional file 1: Suppl. Table 1.** Information of components in Bupi Yishen Formula (BYF). **Suppl. Item1.** Method of high-performance liquid chromatography in chemical analysis of BYF Extract. **Suppl. Item2.** Reagents used for Liquid chromatography–tandem mass spectrometry. **Suppl. Fig. 1.** HPLC analysis of Bupi Yishen Formula in present study(A) and previous study(B)**.** The denotation peaks 1–7: (1) Calycosin-7-O-Glc, (2) (E)-THSG, (3) Astragulin, (4) Rosmarinic acid, (5) Salvianolic acid A, (6) Salvianolic acid B, (7) Calycosin.

**Additional file 2.**

**Additional file 3.**

## Data Availability

The datasets used and analyzed during the current study are available from the corresponding author on reasonable request.

## Abbreviations

AhR: aryl hydrocarbon receptor
BYF: Bupi Yishen Formula
CKD: chronic kidney disease; HPLC: high-performance liquid chromatography; IL: interleukin; KA: kynurenic acid; KEGG: Kyoto Encyclopedia of Genes and Genomes; MS: mass spectrometry; Nx: nephrectomy; QC: quality control; TCM: traditional Chinese medicine; UHPLC: ultra-high-performance liquid chromatography.
