# Supplementary material for: Bupi Yishen formula attenuates kidney injury in 5/6 nephrectomized rats via the tryptophan-kynurenic acid-aryl hydrocarbon receptor pathway
Source: BMC Complement Med Ther. 2021 Aug 10;21:207. doi: 10.1186/s12906-021-03376-1 (PMC8353787; doi:10.1186/s12906-021-03376-1)
Supplement: Supplementary file 1 — Additional file 1: Suppl. Table 1. Information of components in Bupi Yishen Formula (BYF). Suppl. Item1. Method of high-performance liquid chromatography in chemical analysis of BYF Extract. Suppl. Item2. Reagents used for Liquid chromatography–tandem mass spectrometry. Suppl. Fig. 1. HPLC analysis of Bupi Yishen Formula in present study(A) and previous study(B). The denotation peaks 1–7: (1) Calycosin-7-O-Glc, (2) (E)-THSG, (3) Astragulin, (4) Rosmarinic acid, (5) Salvianolic acid A, (6) Salvianolic acid B, (7) Calycosin. [file 12906_2021_3376_MOESM1_ESM.docx]

Supplementary Material

**Suppl. Table 1.** **Information of components in Bupi Yishen Formula (BYF).**

| **Chinese Pin Yin name** | **Latin Binomial Name** | **English name** | **Medical Parts** | **Place of Origin (Province)** | **Lot.No** |
| --- | --- | --- | --- | --- | --- |
| Huangqi | *Astragali Radix* | *Astragalus mongholicus* | Root | Gansu | 190400391 |
| Dangshen | *Codonopsitis Radix* | *Codonopsis pilosula* | Root | Gansu | 190403121 |
| Baizhu | *Atractylodis Macrocephalae Rhizoma* | *Atractylodes macrocephala* | Stem | Anhui | 190100159 |
| Fuling | *Poria* | *Poria cocos* | Sclerotium | Hunan | 190405151 |
| Shanyao | *Diosscoreae Rhizoma* | *Dioscorea opposita* | Root | Henan | 190504491 |
| Yiyiren | *Coix seed* | *Coicis Semen* | Seed | Guizhou | 190404531 |
| Heshouwu | *Polygoni Multiflori Radix* | *Polygonum multiflorum* | Root | Guangdong | 190505051 |
| Tusizi | *Cuscutae Semen* | *Cuscuta Chinensis* | Seed | Shandong | 190405491 |
| Danshen | *Miltiorrhizae Radix Salvia* | *Salvia miltiorrhiza* | Root | Jiangsu | 190505081 |

**Suppl. Item1: Method of high-performance liquid chromatography in chemical analysis of BYF Extract**

The qualitative and quantitative analysis of BYF was performed on a Thermo Accela UHPLC system (San Joes, USA) with a diode-array detector (DAD). The LC separation was conducted on a phenomenex Kinetex C18 column (2.1×100mm, 1.7µm). The mobile phase consisted of water (A) and acetonitrile (B), both containing 0.1% formic acid. The elution gradient was set as follows: 0–12min (10–25% B), 12– 25min (25–32% B), 15–42min (32–56% B), 42–51min (56–95% B). The injection volume of samples was 2 µL with a ﬂow rate of mobile phase at 200 µL/min. Acetonitrile (A996-4) were purchased from Thermo Fisher Scientific (USA). Formic acid was purchased from DIMKA (50144-50ml, USA) and ammonium formate (17843-250G，Honeywell Fluka, USA) was obtained from Fluka.

**Suppl. Item2：Reagents used for Liquid chromatography–tandem mass spectrometry.**

MS-grade methanol (A454-4) and acetonitrile (A996-4) were purchased from Thermo Fisher Scientific (USA). Formic acid was purchased from DIMKA (50144-50ml, USA) and ammonium formate (17843-250G，Honeywell Fluka, USA) was obtained from Fluka. Ultrapure water was filtered through the Milli-Q system. The internal standard mix 1 (IS1) contains: D3-L-Methionine (100 ppm, TRC, Canada), 13C9-Phenylalanine (100ppm, CIL, USA), D6-L-2-Aminobutyric Acid(100ppm, TRC, Canada), D4-L-Alanine (100ppm, TRC, Canada), 13C4-L-Threonine (100ppm, CIL, USA), D3-L-Aspartic Acid (100ppm, TRC, Canada), 13C6-L-Arginine (100ppm, CIL, USA). The internal standard mix 2 (IS2) was purchased from Avanti (SPLASH^TM^ Lipidomix Mass Spec Standard, 330707, Avanti Polar Lipids, USA), which contains LPC 18:1(d7), 25 μg/mL; LPE 18:1(d7), 5 μg/mL; PC 15:0–18:1(d7), 160 μg/mL; PE 15:0–18:1(d7), 5 μg/mL; PG 15:0–18:1(d7), 30 μg/mL; PS 15:0–18:1(d7), 5 μg/mL; PI 15:0–18:1(d7), 10 μg/mL; PA 15:0–18:1(d7), 7 μg/mL; SM d18:1–18:1(d9), 30 μg/mL; cholesterol(d7), 100 μg/mL; CE 18:1(d7), 350 μg/mL; MG 18:1(d7), 2 μg/mL; DG 15:0–18:1(d7), 10μg/mL; and TG 15:0–18:1(d7)–15:0, 55 μg/mL.





Suppl. Figure 1: HPLC analysis of Bupi Yishen Formula in present study(A) and previous study(B)**.** The denotation peaks 1-7: (1) Calycosin-7-O-Glc, (2) (E)-THSG, (3) Astragulin, (4) Rosmarinic acid, (5) Salvianolic acid A, (6) Salvianolic acid B, (7) Calycosin.
