# Supplementary figures and images for: Bupi Yishen formula attenuates kidney injury in 5/6 nephrectomized rats via the tryptophan-kynurenic acid-aryl hydrocarbon receptor pathway
Source: BMC Complement Med Ther. 2021 Aug 10;21:207. doi: 10.1186/s12906-021-03376-1 (PMC8353787; doi:10.1186/s12906-021-03376-1)

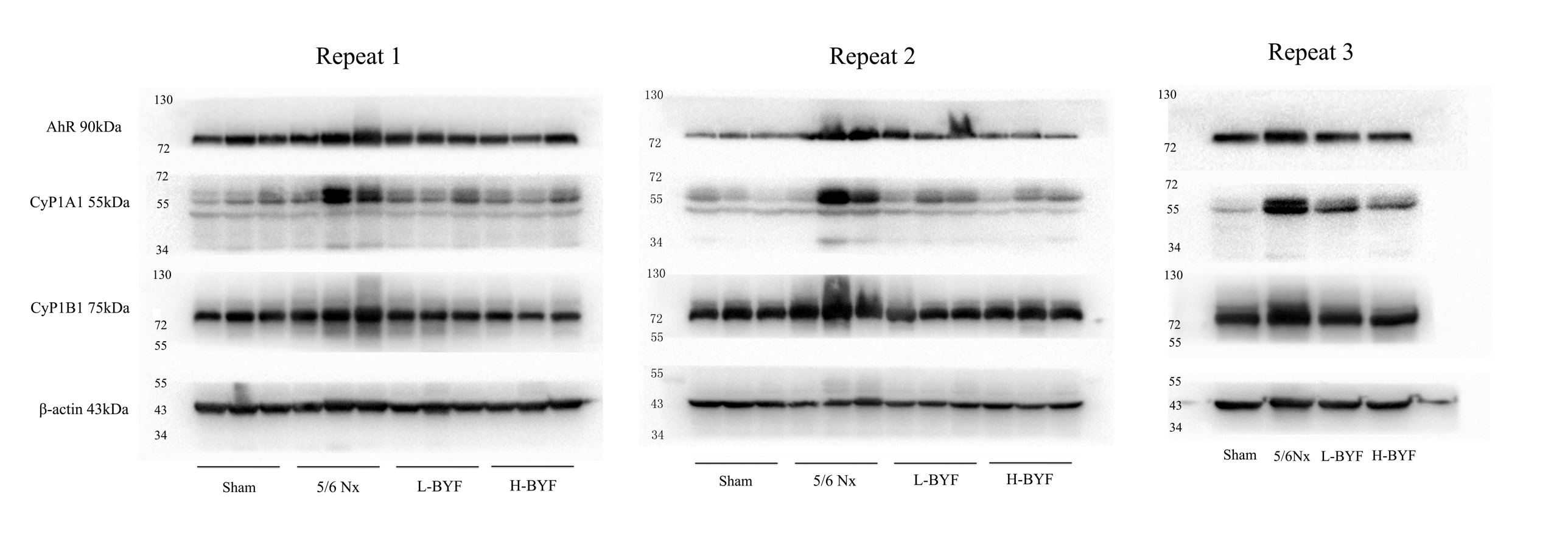

Supplement: Supplementary file 2 — Additional file 2. [file 12906_2021_3376_MOESM2_ESM.tif]
